# Ultrasonic Measurements on β Cyclodextrin/Hydroxyapatite Composites for Potential Water Depollution

**DOI:** 10.3390/ma10060681

**Published:** 2017-06-21

**Authors:** Daniela Predoi, Mihai Valentin Predoi, Simona Liliana Iconaru, Moncef Ech Cherif El Kettani, Damien Leduc, Alina Mihaela Prodan

**Affiliations:** 1 National Institute of Materials Physics, 405A Atomistilor Street, P.O. Box MG7, Magurele 077125, Romania; simonaiconaru@gmail.com; 2 University Politehnica of Bucharest, BN 002, 313 Splaiul Independentei, Sector 6, Bucharest 060042, Romania; predoi@gmail.com; 3 Université du Havre, 75 rue Bellot, CS 80 540, 76058 Le Havre, France; elkettani@univ-lehavre.fr (M.E.C.E.K.); damien.leduc@univ-lehavre.fr (D.L.); 4 Emergency Hospital Floreasca Bucharest, 8 Calea Floresca, Sector 1, Bucharest 014461, Romania; prodan1084@gmail.com; 5 Carol Davila University of Medicine and Pharmacy, 8 Eroii Sanitari, Sector 5, Bucharest 050474, Romania

**Keywords:** hydroxyapatite, ultrasonic measurements, water depollution, lead toxicity

## Abstract

This paper presents structural, morphological and preliminary ultrasonic characterizations of the β-Cyclodextrin/hydroxyapatite (CD-HAp) composites synthesized by an adapted co-precipitation method. The structural and morphological properties were evaluated by Scanning Electron Microscopy (SEM) and Energy Dispersive X-ray Spectroscopy (EDX). The specific surface area, pore size and pore volume were determined using the methods of Brunauer–Emmett–Teller (BET) and Barrett–Joyner–Halenda (BJH), respectively. The novelty of our study consists in preliminary ultrasonic measurements conducted on CD-HAp composite, uniformly dispersed in distilled water. The benefit of this non-destructive method was to facilitate and simplify the characterization techniques of nanoparticles. Our experiments proved that the efficiency of lead ion removal by CD-HAp composites depended on the initial concentration of lead. The maximum adsorption capacity of the solid phase, for Pb^2+^ indicated a higher rate of removal by the CD-HAp_2. These adsorption results bring valuable insight into the beneficial contribution of our compounds, for the removal of heavy metal ions from aqueous solutions. Furthermore, in the present study, was evaluated the toxic effect of lead ions adsorbed by hydroxyapatite from contaminated water on HeLa cells.

## 1. Introduction

In a world where advances are constantly made in a number of fields such as medicine, engineering or robotics, there are still pressing issues that must be dealt with. One of the major global concerns refers to the pollution of water supplies. Water is vital for human survival, considering that almost 70% of the weight of an average adult is comprised of water. From an environmental point of view, Earth’s water supplies are limited. Due to the progress made in the medical field, the life expectancy of humans has increased, a study made by the US National Institute of Aging concluding that it had “increased dramatically” during the 20th century. In this context, the demand for clean water has also reached very high levels [1,2]. Therefore, the goal of scientists worldwide is to create and develop innovative materials capable of efficiently removing pollutants from waters. Currently, there are a great variety of methods used for the removal of inorganic pollutants from waters, among which, the most popular are the precipitation method, ion exchange method, adsorption method, bio-membrane filtration, reverse osmosis or electrochemical precipitation [1,3,4,5,6,7]. A material whose properties make it a promising candidate for potential applications for environmental depollution is hydroxyapatite (HAp) [1,8,9,10,11]. Being very similar to the inorganic part of the human hard tissue, synthetic hydroxyapatite has been widely used for a number of biomedical applications [12,13,14,15,16,17,18,19,20,21,22,23,24]. Nowadays, due to its biocompatibile and osteoconductive properties, HAp is being used for the reconstruction and regeneration of damaged bone tissue, or as a covering material for metallic implants [1,12,17,25]. On the other hand, there are studies [1,10,26,27] that describe the potential of hydroxyapatite as an active agent in the removal of various heavy metals from water and soil. Due to the relatively reduced costs involving the synthesis of HAp, a number of studies [1,28,29] have demonstrated that HAp could be efficient for the removal of Pb^2+^ ions from polluted waters and soils.

Among the materials used for their potential applications for depollution of aqueous media without having negative influences on the biological life [30,31,32], hydroxyapatite has the advantage of exhibiting a hexagonal structure which is identical to bone apatite.

On the other hand, cyclodextrines are cyclic oligosaccharides, soluble in water, which are comprised of minimum six [1,2,3,4] linked α-d-glucosyl residues [12]. Among cyclodextrines, β-Cyclodextrin (β-CD) is a cyclic oligosaccharide with seven α-(1,4) linked glycosyl groups in its macrocyclic structures [33,34]. Having a doughnut-like shape, it has the ability to incorporate hydrophobic moieties/molecules in the cavity [33,35].

Considering the properties possess by both HAp and β-CD, with respect to potential environmental applications, this paper is focused on the study of a compound based on HAp and two different concentrations of β-CD (CD-HAp_1 and CD-HAp_2). To this end, the colloidal characteristics of aqueous solutions were analyzed by Dynamic Light Scattering (DLS) and zeta potential, while the morphological investigations were performed by scanning electron microscopy (SEM). Furthermore, to better characterize the CD-HAp_1 and CD-HAp_2 compounds, a non-destructive method was used. Ultrasonic measurements have been recently used for the characterization of particles dispersions, but this field is just starting to develop [36,37,38,39,40]. Thus, ultrasonic measurements were performed on the CD-HAp_1 and CD-HAp_2 dispersions and valuable information on the material properties were acquired. The novelty of this study consists in the results obtained upon adsorption experiments of lead ions by CD-HAp_1 and CD-HAp_2 compounds as well as the new material characterization approach involving ultrasonic measurements. The contribution of our research could bring valuable information to the researchers in this field, and improve the study of nanoparticles using ultrasonic measurements. The benefit of this non-destructive method consists in a modern and rapid characterization of nanometric particles. Furthermore, given the fact that there is a global interest in finding new water depollution methods, our experimental results regarding the adsorption of lead ions from aqueous solutions could bring a substantial contribution to the community of scientists devoted to this environmental problem. Moreover, the evaluation of toxic effects of lead ions on HeLa cells, highlights the effectiveness of CD-HAp composite in depollution of contaminated water, which can lead to the reduction of this major risk to public health.

## 2. Results

The goal of this research was to prepare β-Cyclodextrin/hydroxyapatite (CD-HAp) composite by an adapted precipitation method [37] with the Ca/P stoichiometric ratio maintained at 1.67 which could be used as a new an alternative adsorbent for removal of lead ions from contaminated water. The Ca(NO_3_)_2_·4H_2_O solution was added in 50 mL (CD-1) and 100 mL (CD-2) of β-CD suspension under constant stirring. The (NH_4_)_2_HPO_4_ solution was added drop by drop in the solution based on Ca(NO_3_)_2_·4H_2_O and β-Cyclodextrin. The CD-HAp_1 and CD-HAp_2 nanocomposites were obtained redispersing the CD-HAp composites obtained after washing with deionized water and centrifuging in 50 mL and 100 mL of β-CD under constant stirring for 72 h. Moreover, complex characterizations of the CD-HAp_1 and CD-HAp_2 nanocomposites were conducted by Dynamic Light Scattering (DLS) and zeta potential, ultrasonic measurements, N_2_ adsorption/desorption, scanning electron microscopy (SEM), and the Flame Atomic Absorption Spectrometry (AAS). Besides, the effect of CD-HAp nanocomposites on HeLa cells and effect of lead ions on HeLa cells morphology were also investigated.

**Figure 1:**
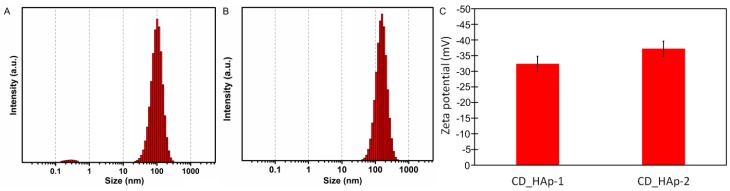
Particle size distribution measured by DLS of: CD-HAp_1 (**A**); and CD-HAp_2 (**B**) nanoparticles suspensions. Zeta potential of CD-HAp_1 and CD-HAp_2 nanoparticles suspensions (**C**).

The colloidal characteristics of CD-HAp_1 and CD-HAp_2 nanoparticles suspensions were analyzed by Dynamic Light Scattering (DLS) and zeta potential (Figure 1). Prior to measuring the zeta potential and particle sizes, the solutions were subjected to ultrasonic agitation for 30 min. The intensity of size distribution was computed by the method of regularization with a software of SZ-100 Nanoparticle Analyzer, depending on the hydrodynamic radius, R_h_, estimated using the Stokes–Einstein equation:

(1) $$R_{h}=\frac{kT}{6\pi {ƞ}_{0}D_{0}}$$

where *k* is the Boltzmann constant, *T* is the absolute temperature, ƞ_0_ is the viscosity of the solvent and *D*_0_ is the diffusion coefficient at infinite dilution.

The particle size distribution measured by DLS of CD-HAp_1 and CD-HAp_2 samples is presented in Figure 1A,B. From the analysis of particle size distributions, it can be observed that the CD-HAp_2 sample presented a unimodal population with narrow particle size distribution about 163 ± 2 nm. The CD-HAp_1 sample revealed a bimodal distribution. The first population containing a few dozen particles with a particle size distribution about 0.27 nm is not important for the total quantity. According to Bonini et al. [41], the development of discrete aggregates at lower concentrations could be favored by the formation of hydrogen bonds. The particle size distribution of CD-HAp_1 sample obtained by DLS was 97.7 ± 4 nm. Furthermore, it can be seen that in the CD-HAp_1 nanocomposites the aggregation was minimal. The results obtained from DLS studies for the CD-HAp_1 and CD-HAp_2 samples are in good agreement with those previously obtained by Lucio et al. [42]. Moreover, Wang et al. [43], in their previous studies based on β-cyclodextrin, modified magnetic graphene oxide nanocomposites.

Besides, zeta potential values of the CD-HAp_1 and CD-HAp_2 nanocomposites were also measured. The zeta potential of the samples is shown in Figure 1C. It can be seen that the average zeta potential of the particles of both samples is negative. The zeta potential value of −32.4 mV was found for CD-HAp_1 nanocomposites. The zeta potential value increased to around −37.2 mV for CD-HAp_2 nanocomposites, which could prove a better stability and dispersion of CD-HAp_2 nanocomposites. According to previous studies [44,45], the stabilization of CD-HAp_2 nanocomposites in solution could be attributed to intermolecular cross-linkages that are formed under ambient conditions.

**Figure 2:**
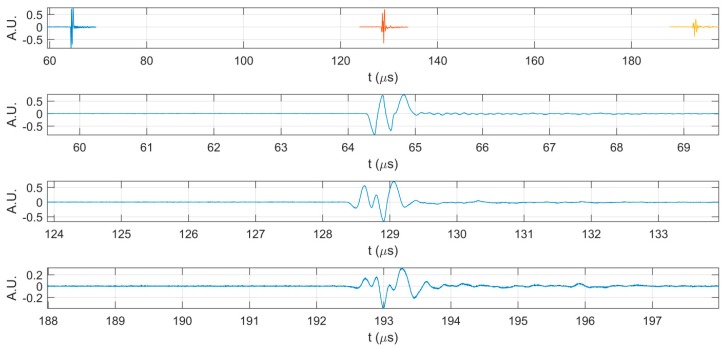
The first three echoes acquired for double-distilled water: together (**top**); and separated (**below**).

Additional information on the behavior of CD-HAp_1 and CD-HAp_2 dispersions was obtained by ultrasonic non-destructive measurements. Specific parameters, such as ultrasonic velocity and attenuation coefficients, were determined for all three samples at the same temperature (21 °C), for all measured solutions. In Figure 2, are presented the first three echoes acquired for the double-distilled water, which was considered as reference for the other two solutions (CD-HAp_1 and CD-HAp_2). All experimental data were obtained using a transducer with a central frequency of f = 5 MHz. The ultrasonic velocity for the reference, namely double-distilled water, was 1492.07 m/s and the attenuation coefficient was 0.936 (Np/m).

**Figure 3:**
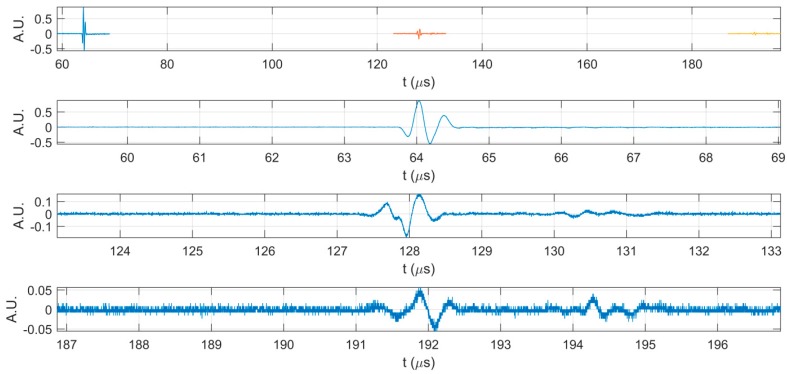
The first three echoes acquired for CD-HAp_1 sample: together (**top**); and separated (**below**).

**Figure 4:**
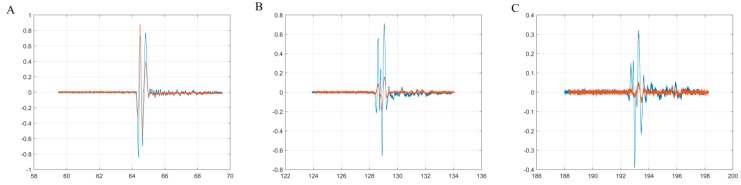
Detailed comparison between the first three echoes registered for double-distilled water (blue) and CD-HAp_1 solution (orange). First echo (**A**), second echo (**B**) and third echo (**C**).

In Figure 3 are highlighted the first three echoes acquired for CD-HAp_1 solution. Figure 4 presents detailed comparisons between the echoes registered for the reference and the CD-HAp_1 solution. Time differences between each of the three echoes, recorded for bi-distilled water on the one hand, and the dispersion based on CD-HAp_1 on the other hand, were determined. Therefore, in the case of the first echo (Figure 4A), the time difference between the bi-distilled water (blue) and the CD-HAp_1 dispersion (orange) was 0.467 μs.

For the second echo (Figure 4B), the time difference was 0.936 μs, leading to a relative time difference, for one way propagation, between the signals of 0.468 μs. Lastly, in the case of the third echo (Figure 4C), the time difference was 1.163 μs, which corresponded to a relative time difference of 0.388 μs. Considering these time differences and the distance covered by the signals, the ultrasound velocity for the CD-HAp_1 was determined: 1502.39 ± 1.09 (m/s), with an attenuation of 7.965 Np/m.

**Figure 5:**
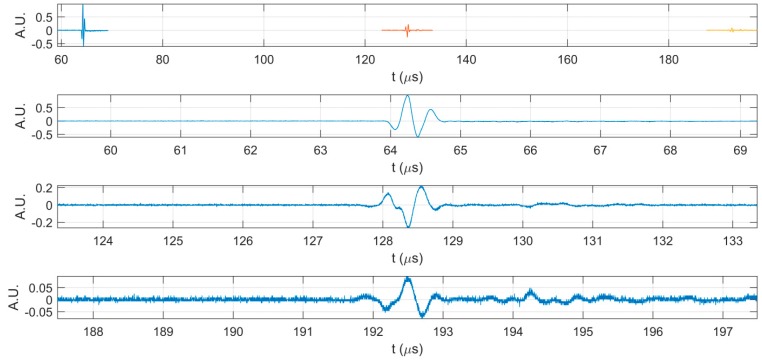
The first three echoes acquired for CD-HAp_2 sample.

**Figure 6:**
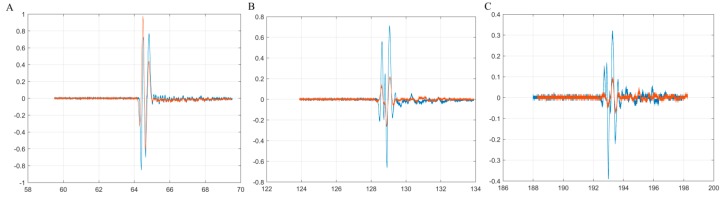
Detailed comparison between the first three echoes registered for double-distilled water (blue) and CD-HAp_2 solution (orange). First echo (**A**), second echo (**B**) and third echo (**C**).

Similar to the case if CD-HAp_1 dispersion, in Figure 5 are presented the first three echoes acquired for the CD-HAp_2 dispersion, while in Figure 6 are depicted the detailed comparisons made between the first three echoes associated to double-distilled water (blue) and CD-HAp_2 dispersion (orange). The time difference calculated for the first echo (Figure 6A) was 0.264 μs. For the second echo (Figure 6B), the time difference was 0.532 μs, thus leading to a relative time difference of 0.266 μs. In the case of the third echo (Figure 6C), the time difference was 0.788 μs, which corresponds to a relative time difference of 0.263 μs. Therefore, given these time differences, the ultrasound velocity obtained for the CD-HAp_2 dispersion was 1498.24 ± 0.04 (m/s), and the attenuation, 6.84 Np/m.

Due to the increased attenuation of ultrasonic waves through the CD-HAp_1 and CD-HAp_2 dispersions, other weak signals associated to the reflections from the cup bottom disappeared, or diminished drastically, compared to the signals obtained for double-distilled water (Figure 2 and Figure 6). Moreover, the ultrasonic measurements allowed the estimation of the particle size distribution. The principle of measuring the particle size was based on the attenuation of the ultrasound waves at different frequencies, traveling through dispersions containing particles of different sizes [46]. According to previous studies [46], the estimation of the particle size distribution was done by measuring sound energy loss at multiple frequency values. The mean particle size of CD-HAp_1 and CD-HAp_2 samples determined by ultrasonic measurements was 23.5 ± 2 nm and 16.8 ± 4 nm, respectively.

**Figure 7:**
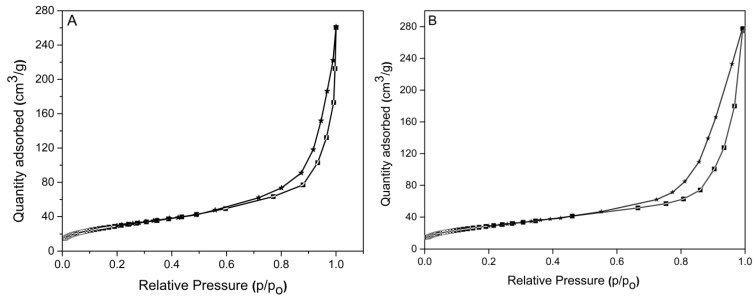
The nitrogen adsorption/desorption isotherms of: CD-HAp_1 (**A**); and CD-HAp_2 (**B**) nanocomposites.

The nitrogen adsorption/desorption isotherms of CD-HAp_1 and CD-HAp_2 nanocomposites are presented in Figure 7A,B. It can be seen that the respective N_2_ adsorption/desorption isotherms of CD-HAp_1 and CD-HAp_2 nanocomposites exhibited typical type IV isotherms according to the International Union of Pure and Applied Chemistry (IUPAC) classification on the basis of size, as mesopores in the range of 2 nm–50 nm [47]. The synthesized CD-HAp_1 and CD-HAp_2 samples presented a distinct hysteresis loop, indicating that the samples have properties of typical mesoporous materials [47]. The Barrett–Joyner–Halenda (BJH) method was used to determine the total pore volume V_p_ and pore size S_p_ of CD-HAp_1 and CD-HAp_2 nanocomposites. The values for the BET specific surface area (S_BET_), the total pore volume (V_p_) and pore size (D_p_) are listed in Table 1.

The Langmuir and Freundlich desorption isotherm models were used to fit the experimental data of Pb^2+^ sorption onto CD-HAp_1 and CD-HAp_2. The adsorption isotherms and adsorption kinetics experiments have been conducted by varying the concentrations of Pb^2+^ in the studied solutions, maintaining all the other experimental conditions. All the experiments were realized in triplicate and the amounts of Pb^2+^ ions adsorbed per unit mass of CD-HAp_1 and CD-HAp_2, (q_e_), were calculated according to Cho et al. [48]:

(2) $$q_{e}=\frac{V\left(C_{o}-C_{e}\right)}{W}$$

where *C_o_* and *C_e_* were the initial and equilibrium concentrations of adsorbate (mg/L), *W* was the dry mass of the adsorbent (g), and *V* was volume of the solution (L). The experimental data were fitted by the well-known Langmuir function [49]:

(3) $$\frac{C_{e}}{q_{e}}=\frac{C_{e}}{q_{m}}+\frac{1}{K_{L}\times q_{m}}$$

where *C_e_* (mg/L) was the equilibrium concentration of the adsorbate, *q_e_* (mg/g) was the equilibrium adsorption capacity of the adsorbents, *q_m_* (mg/g) was the saturated adsorption capacity and *K_L_* is Langmuir constant. To determine the Freundlich constant, related to the adsorption capacity and the constant related to adsorption density, the linear form of the Freundlich equation [50] was used:

(4) lnqe=lnKF+1nlnCe

where *q_e_* is the amount adsorbed at equilibrium concentration (mg/g), *K_F_* is the empirical constant of Freundlich isotherm (L/mg), *C_e_* is the equilibrium concentration of lead ions in solution (mg/L) and *n* is an empirical parameter related to the intensity of adsorption.

**Figure 8:**
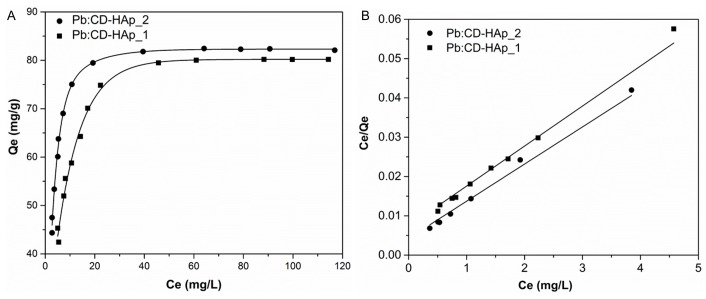
Equilibrium adsorption isotherms for Pb^2+^ onto CD-HAp_1 and CD-HAp_2 at room temperature (**A**); and the linearized Langmuir fit (**B**).

For a good evaluation of the adsorption performances of CD-HAp_1 and CD-HAp_2, the amounts of adsorbed lead as a function of lead concentrations at equilibrium were plotted (Figure 8A). On the other hand, the linearized Langmuir fit for the adsorption isotherms of Pb^2+^ was presented (Figure 8B).

At room temperature, the correlation coefficient of Langmuir isotherm (R^2^) for Pb^2+^ removal by CD-HAp_2 had a higher value than in the case of Pb^2+^ by CD-HAp_1. On the other hand, the maximum adsorption capacity for the solid phase, *q_m_* (mg/g), for Pb^2+^ indicated a higher rate of removal of Pb^2+^ by CD-HAp_2. The linear plot of *C_e_*/*q_e_* against *C_e_* exhibited in Figure 8B revealed that the adsorption is in proper agreement with the Langmuir model. The correlation coefficient, R^2^, was equal to 0.99 for CD-HAp_1 and 0.992 for CD-HAp_2 while the Langmuir constant *K_L_* for the adsorption of Pb^2+^ was 1.378 L/mg for CD-HAp_1 and 2.218 L/mg for CD-HAp_2. Moreover, the maximum adsorption capacity *q*_max_ were equal to 98.232 mg/g for CD-HAp_1 and 105.820 mg/g for CD-HAp_2. In agreement with previous studies [51,52] on removal of lead and zinc ions from water by low cost adsorbents, such as granular bentonite used in the removing of mercury (II), cadmium (II) and lead (II) from aqueous solutions, the Freundlich isotherm model has proven to be good for heterogeneous surfaces.

**Figure 9:**
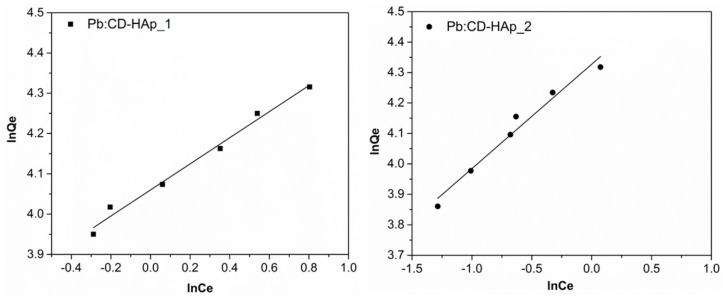
Freundlich linearized fits for the adsorption of lead CD-HAp_1 and CD-HAp_2 samples.

On the other hand, the Freundlich isotherm model provides information on increasing the concentration of ionic adsorbed species on the surface of the solid, when the concentration of said species in the liquid phase is increased. Fitting the data using the Freundlich adsorption isotherm model (Figure 9), a linear relationship has been obtained with *K_F_* equal to 57.971 for CD-HAp_1 and 75.733 for CD-HAp_2, while the 1/n was equal to 0.324 for CD-HAp_1 and 0.343 for CD-HAp_2. The factor R was equal to 0.981 for CD-HAp_1 and 0.984 for CD-HAp_2. Following these results, it can be seen that the inverse of the empirical parameter related to the intensity of adsorption has values in the range 0.1 < 1/n < 1, which proves that the adsorption process was in good agreement with previous studies [53]. Table 2 lists the parameters for the adsorption capacity data of CD-HAp_1 and CD-HAp_2 after fitting the experimental data.

The results obtained showed that the value of K_F_ constant is higher for CD-HAp_2 sample and the 1/n value of CD-HAp_2 sample is smaller than that of CD-HAp_1 sample. This shows that the binding capacity in this case was of higher and the affinity between the adsorbent and lead ions is also higher than for CD-HAp_1 sample. Furthermore, the correlation coefficients, R^2^, presented in Table 2, revealed that the Langmuir equation offers a better fit than Freundlich equation for adsorption isotherms.

**Figure 10:**
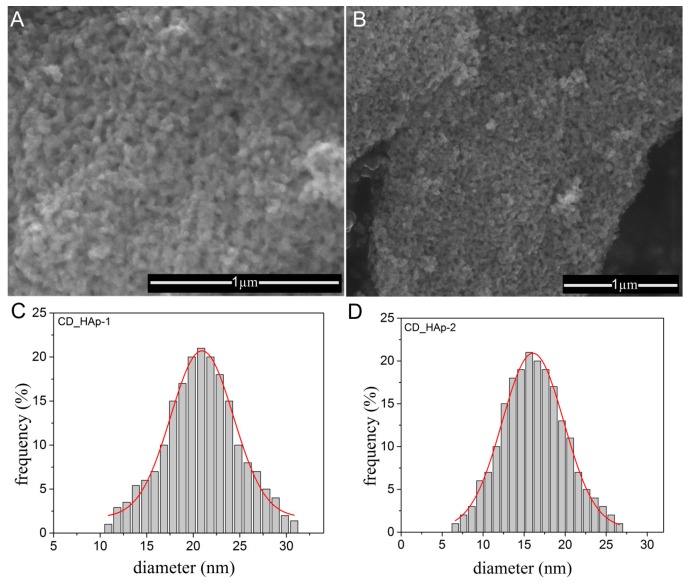
SEM images of: CD-HAp_1 (**A**); and CD-HAp_2 (**B**) samples before adsorption of lead ions. The mean particle size of: CD-HAp_1 (**C**); and CD-HAp_2 (**D**) samples.

The surface appearance of CD-HAp_1 and CD-HAp_2 samples before and after the adsorption of lead ions were analyzed for comparison. The SEM images obtained for the CD-HAp_1 and CD-HAp_2 samples before adsorption of lead ions are presented in Figure 10A,B.

The agglomerated particles with homogenous size were observed. The increase of the β-cyclodextrin amount in the samples did not lead to major changes in the morphology of the particles, but a change in the particles dimension was observed (Figure 10C,D), in good agreement with the DLS studies.

**Figure 11:**
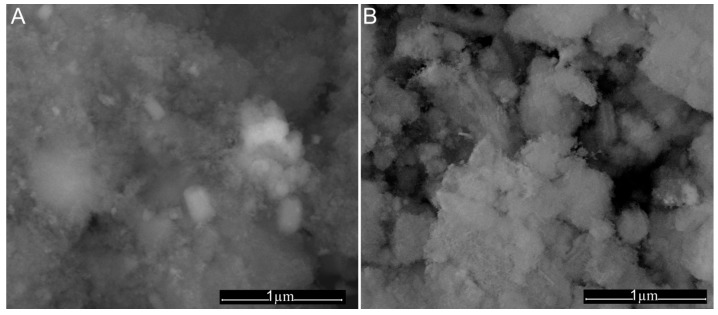
SEM images of: CD-HAp_1 (**A**); and CD-HAp_2 (**B**) samples after the adsorption of Pb^2+^.

The mean particle size of CD-HAp_1 and CD-HAp_2 samples was estimated to 20.8 ± 1 nm and 15.2 ± 2 nm, respectively. The surface morphology of CD-HAp_1 and CD-HAp_2 samples changed after the adsorption of lead ions (Figure 11A,B). It can be seen that flake-like species were formed on the surface of CD-HAp_1 and CD-HAp_2 samples after the adsorption of Pb^2+^.

The comparison of mean particle size resulting from DLS, SEM studies and ultrasonic (US) measurements were presented in Table 3.

**Figure 12:**
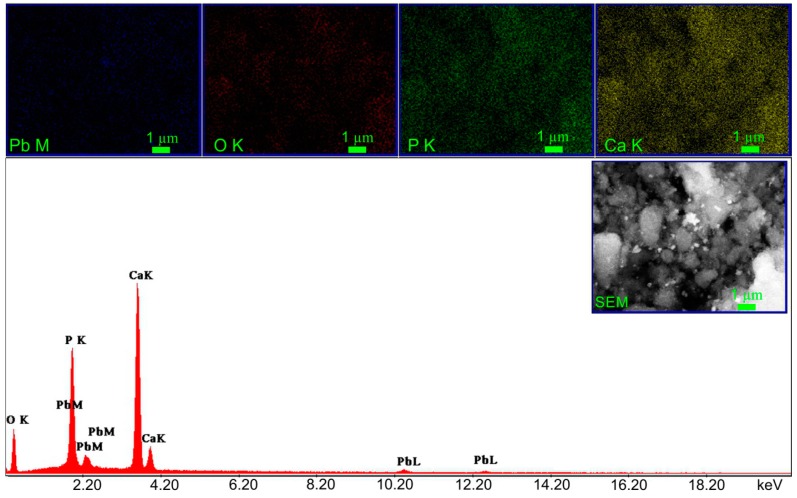
EDX spectrum and elemental mapping of CD-HAp_1 sample after the adsorption of Pb^2+^.

**Figure 13:**
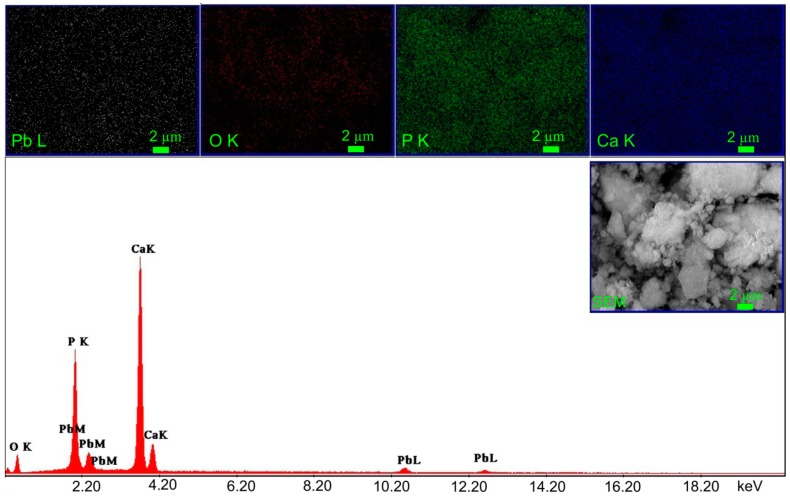
EDX spectrum and elemental mapping of CD-HAp_2 sample after the adsorption of Pb^2+^.

The EDX spectra and elemental mapping of CD-HAp_1 and CD-HAp_2 samples, after the adsorption of lead ions, are presented in Figure 12 and Figure 13. The obtained EDX spectra for the investigated nanocomposites highlighted the presence of peaks for Ca, P and O, which are the major constituent elements of hydroxyapatite. The peaks attributed to Pb^2+^ element appeared in the EDX spectra also. The strong peaks at 2.34 and 10.5 keV in the EDX spectra represent a clear evidence of lead ion adsorption onto the surface of CD-HAp_1 and CD-HAp_2 samples. Furthermore, the peaks attributed to lead element were more intense in the EDX spectra of CD-HAp_2 samples. These results clearly show that lead ions were efficiently adsorbed onto the surface of both samples. Besides, the elemental mapping of CD-HAp_1 and CD-HAp_2 samples after the adsorption of lead ions exhibited in Figure 12 and Figure 13 provided information about the uniform distribution of the elements through the samples and their homogeneity.

**Figure 14:**
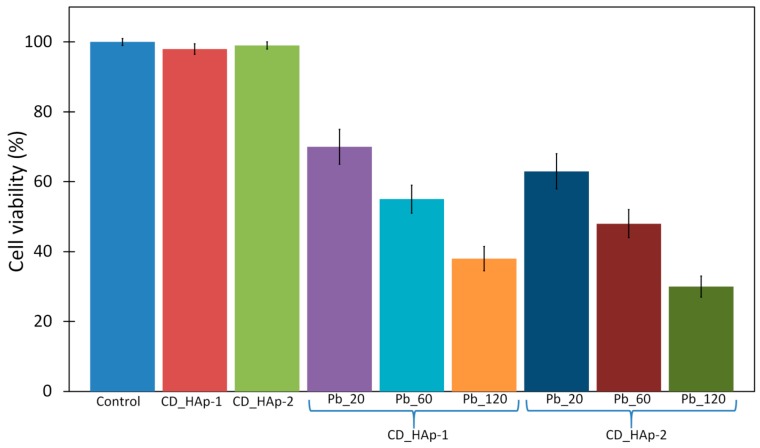
Cell viability assay in HeLa cells for CD-HAp_1 and CD-HAp_2 before and after the adsorption of lead ions (20, 60 and 120 mg/L) for 24 h of incubation at 37 °C. Each value represents the mean value ± SD (*** *p* < 0.001, *n* = 4).

**Figure 15:**
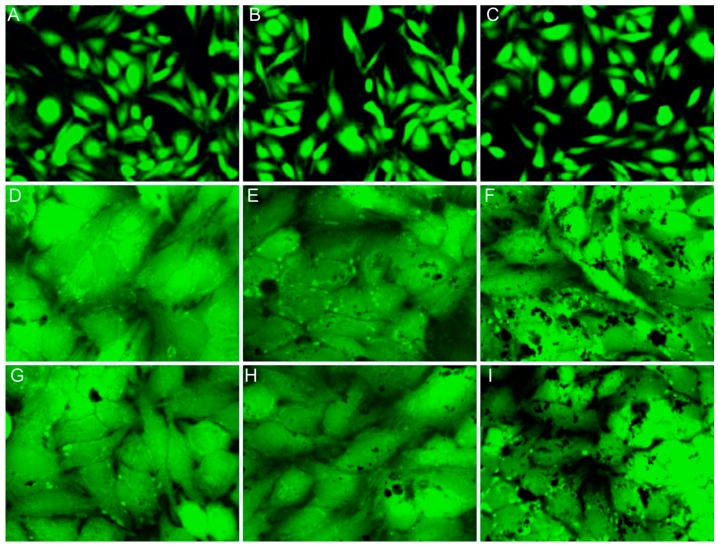
Effect of lead ions on HeLa cells morphology: control cells (**A**); HeLa cells with the: CD-HAp_1 (**B**); and CD-HAp_2 (**C**) nanocomposite; HeLa cells with the CD-HAp_1 nanocomposite after the adsorption of: 20 mg/L (**D**); 60 mg/L (**E**); and 120 mg/L (**F**) lead ions; and HeLa cells with the CD-HAp_2 nanocomposite after the adsorption: of 20 mg/L (**G**); 60 mg/L (**H**); and 120 mg/L (**I**) lead ions. (×200).

The in vitro cytotoxicity of the CD_HAp-1 and CD_HAP-2 samples before and after immersion in contaminated solutions, with different lead concentrations was evaluated using the MTT viability assay, against HeLa cell lines. As can be seen in Figure 14, the CD_HAp-1 and CD_HAP-2 nanocomposites had no cytotoxicity and showed the same behavior as the control sample for the time duration of 24 h, indicating a good biocompatibility. On the other hand, for the CD_HAp-1 sample after the adsorption of Pb^2+^ (20 mg/L) was observed a low cytotoxicity after 24 h of incubation in the presence of HeLa cells. From Figure 14 it can be seen that the cytotoxicity of the CD_HAp-1 and CD_HAp-2 samples is strongly influenced by the amount of lead ions in the contaminated solution. Furthermore, the CD_HAp-1 and CD_HAp-2 samples after the adsorption of Pb^2+^ (60 mg/L) exhibited high cytotoxicity in HeLa cells line. This may be due to the adsorption of lead ions from contaminated solutions. The results obtained for the samples examined after the adsorption of lead ions from the most contaminated solutions (120 mg/L) showed the highest cytotoxicity. These results revealed that the lead ions present in the recovered samples after decontamination of solutions, play an important role in decreasing cell viability. The microscopic images of HeLa cells after 24 h incubation with CD-HAp composites before and after the adsorption of lead ions corroborate with the MTT determinations (Figure 15). The HeLa cells morphology with CD_HAp-1 and CD_HAp-2 nanocomposites, before adsorption of lead ions (Figure 15B,C) is similar to control cells (Figure 15A).

The HeLa cells treated with CD-HAp_1 and CD-HAp_2 nanocomposites after adsorption of lead ions from contaminated solutions showed a changed morphology. Figure 15D–I exhibited the modifications of HeLa cell morphology, observed in samples that adsorbed lead from contaminated solutions from 20 mg/L to 120 mg/L. The maximum of toxicity was observed for the HeLa cells in the presence of CD-HAp nanocomposites, after adsorption of 120 mg/L lead from contaminated solutions (Figure 15F,I). It can be seen that the morphology of HeLa cells in the presence of CD-HAp_2 nanocomposites, after adsorption of 120 mg/L lead from contaminated solutions, has been severely affected (Figure 15I). It can be concluded that the cell morphology has changed, depending on the decrement of HeLa cell viability.

## 3. Discussion

It is well-known that in the environmental monitoring process, the need for reliable analyzes of particle size and dispersion in soil, water or air is imperative for effective control of the development of new materials. In addition to this, the lead pollution of natural water represents one of the most important problems concerning the human health or aquatic animals, as a result of its accumulation in the food chain. In this context, our study regarding the adsorption of heavy metal pollutants such as lead, from contaminated solutions brings new information on the development of effective adsorbents that have the ability to remove heavy metals from aqueous solutions. The study of particle size and dispersion in different environments is essential for the understanding of colloidal systems, in both synthetic and biological processes. In this context, the use of ultrasound measurements appears to offer a good perspective and a viable alternative, being a non-destructive analysis technique. According to previous studies, the application areas of ultrasonic measurements are dominated by environmental analyzes [54]. The techniques using ultrasonic measurements are non-destructive and non-perturbative to the examined medium, and thereby useful for kinetics studies and in situ monitoring of events.

In order to characterize particle dispersions, the ultrasonic measurements shown great potential as a result of their penetration of opaque systems (particles at high concentration in solution) and the possibility of identifying particles of diameters in a wide range, from tens of nanometers to millimeters. The results of the present research have shown that the average diameters estimated by ultrasonic measurements were comparable with those evaluated by SEM (Table 3). The mean diameter and particle size distribution estimated by DLS were however not identical to those evaluated using the US and SEM methods. The difference between the nanoparticle diameter achieved from the SEM images and the hydrodynamic diameter obtained from the DLS studies can be explained by the fact that in the drying process the particle size decreases by removing the water from the surface. This behavior was in agreement with findings reported by Loh et al. [55]. More than that, in agreement with the DLS and SEM studies, additional information on the behavior of CD-HAp_1 and CD-HAp_2 dispersions, obtained by ultrasonic non-destructive measurements, confirmed that particle size is lower for sample CD-HAp_2, because the calculated attenuation coefficient was higher for sample CD-HAp_2. The adsorption-desorption (nitrogen, N_2_) gas analysis using Brauer–Emmett–Teller (BET) method showed that the CD-HAp_1 and CD-HAp_2 nanocomposites exhibited typical type-IV isotherms, which in turn show that adsorption on mesoporous solids is accomplished via multilayer adsorption, followed by capillary condensation. It has been observed that at higher pressures, the adsorbed amount increases very steeply due to capillary condensation in mesopore. According to previous studies [47], the evaluation of the specific surface area using the BET method is based on the evaluation of the monolayer capacity. The specific surface area of CD-HAp nanocomposites is an important parameter to be evaluated by its porosity. The pore size and pore volume of nanocomposites were in the range of nanometers. The specific surface area of CD-HAp_2 nanocomposites was greater than CD-HAp_1 nanocomposites. It can also be seen that the specific surface area has changed with the increase in the amount of β-Cyclodextrin (Table 1).

Moreover, the in vitro studies on CD-HAp_1 and CD-HAp_2 samples before immersion in contaminated solutions have shown that the CD-HAp composites used for decontamination exhibited a good biocompatibility. On the other hand, the in vitro cell cytotoxicity studies demonstrated that the lead ions presented a high toxicity against HeLa cells. Studies on the toxicological effects of lead ions on HeLa cells showed that they had a morphological change according to previous studies [56,57]. Higher toxicity of the CD-HAp_1 and CD-HAp_2 nanoparticles after the adsorption of 60 mg/L (Figure 15E,H) and 120 mg/L (Figure 15F,I) lead ions in comparison to CD-HAp_1 and CD-HAp_2 nanoparticles after the adsorption of 20 mg/L (Figure 15D,G) counterpart, could be attributed to the higher amount of lead that was adsorbed and the contaminated solution. Moreover, this study demonstrated that the newly obtained adsorbent (CD-HAp), showed excellent adsorbability for Pb^2+^ from aqueous solution. The isotherms parameters for lead adsorption onto CD-HAp_1 and CD-HAp_2 (Table 2), revealed that the Langmuir equation was the most suitable for fitting the adsorption isotherms in accordance with the studies on “adsorptive removal of Pb^2+^, Co^2+^ and Ni^2+^ by hydroxyapatite/chitosan composite from aqueous solution” presented above [58]. On the other hand, the removal efficiency increased with concentration of the β-Cyclodextrin in the sample. According to the studies reported by other authors [59,60], the possible mechanisms involving lead sorption with CD-HAp_1 and CD-HAp_2 nanocomposites should be associated with ion exchange and dissolution-precipitation mechanisms. Even if colloidal dispersions have been analyzed in this paper, the same analytical techniques could be applied for systems of larger particle sizes. It could also be considered that the ultrasound measurements used for the complex analysis of the materials used in this study, will lead to progress in the complex analysis of materials used in various environmental applications.

Finally, we could say that the results of the present research distinctly reveal that CD-HAp nanocomposites could represent an economical source of sorbent for lead ions from aqueous solution and could be used as a new and alternative adsorbent for removal of lead ions from contaminated water.

## 4. Materials and Methods

### 4.1. Materials

In order to synthesize the β-Cyclodextrin/hydroxyapatite (CD-HAp) composite, precursors of calcium nitrate (Ca(NO_3_)_2_·4H_2_O, 99% purity Aldrich, St. Louis, MO, USA), ammonium hydrogen phosphate ((NH_4_)_2_HPO_4_; Wako Pure Chemical Industries Ltd., Richmond, VA, USA) and β-Cyclodextrin (Sigma Aldrich, St. Louis, MO, USA) were used.

### 4.2. Preparation of β-Cyclodextrin/Hydroxyapatite (CD-HAp) Composites

β-Cyclodextrin/hydroxyapatite (CD-HAp) was synthetized by a modified co-precipitation method [37]. Hydroxyapatite was synthesized in air, at a temperature of 80 °C using aqueous solutions. The Ca/P stoichiometric ratio was maintained at 1.67 [37,38]. Appropriate amounts of Ca(NO_3_)_2_·4H_2_O were dissolved in deionized water. A phosphate solution was prepared by dissolving (NH_4_)_2_HPO_4_ in deionized water. The aqueous suspensions of 50 mL of β-CD (CD-1) and 100 mL of β-CD (CD-2) were prepared under magnetic stirring. The Ca(NO_3_)_2_·4H_2_O solution was added in β-CD suspension under constant stirring for 30 min. Finally, the (NH_4_)_2_HPO_4_ solution was added drop by drop under constant stirring on the solution based on β-Cyclodextrin. The resulting CD-HAp composite was filtered, washed with deionized water and centrifuged. After centrifugation, the CD-HAp composite were redispersed in 50 mL (CD-HAp_1) and 100 mL (CD-HAp_2) of β-CD under constant stirring for 72 h. After 72 h of constant stirring, the composite were centrifuged. The 0.4 grams of CD-HAp_1 and CD-HAp_2 composites resulted after centrifugation were redispersed in 40 mL of solution contaminated with lead. The rest of the material obtained was dried in an oven at 80 °C for 72 h.

### 4.3. Adsorption Experiments

Equilibrium adsorption isotherms were carried out on aqueous solutions containing different concentrations of lead. Lead solutions with total metal-ion concentrations in the range of 0.1 to 150 mg/L were prepared by dilution of the stock solutions with deionized H_2_O. Batch adsorption experiments were conducted in silicone tubes of 40 mL. In these experiments, 0.2 g of the adsorbent was used. By addition of 0.1 M hydrochloric acid (HCl) the pH of the solution was maintained at 5. The volume of the solution was maintained at 20 mL, and the mixture was stirred on a Mixer SRT1 Roller for 24 h. After stirring, the tubes were centrifuged at 10,000 rpm for 30 min. The supernatant was filtered. The atomic absorption spectrometry (AAS) was used for analyzing the supernatant which has been filtered in advance. The recovered powders were characterized and the experiments were performed at RT (room temperature).

### 4.4. Physico-Chemical Characterization

The colloidal properties of the CD-HAp_1 and CD-HAp_2 nanocomposites were investigated by Dynamic Light Scattering (DLS) and zeta potential using a SZ-100 Nanoparticle Analyzer Horiba-Jobin-Yvone) at 25 ± 1 °C. All the samples were diluted in distilled water before analysis.

The ultrasonic profiles of the CD-HAp_1 and CD-HAp_2 nanoparticles were acquired by means of a specialized equipment. The measurements were obtained using a H5K model transducer produced by General-Electric, with 5 MHz central frequency and a very short burst. The ultrasonic signals were acquired by a Tektronix DPO 4014B oscilloscope (Tektronix, Inc., Beaverton, OR, USA). The experimental setup was calibrated with double-distilled water at a preset temperature, measured with an accuracy of 0.1 °C. Both samples were measured in the same experimental conditions as the reference.

The porosity of the samples was investigated by adsorption-desorption (nitrogen, N_2_) gas analysis using Brunauer–Emmett–Teller (BET) method. To this end, an ASAP 2020 instrument was used, and the measurements were performed at 77 K.

The Flame Atomic Absorption Spectrometry (AAS) was used for determination of concentration of Pb^2+^ from the aqueous solutions. The AAS measurements were performed using a Zeeman HITACHI Z-8100 from Japan Hitachi (Tokyo, Japan). The AAS measurements were realized under a constant air flow rate and the wavelength used was 283.3 nm according to the operational condition for lead.

In order to investigate the morphology and chemical compositions of the obtained powders (CD-HAp_1 and CD-HAp_2), a Quanta F Inspect scanning electron microscope (SEM) equipped with an EDX/2001 device was used.

### 4.5. Effect of CD-HAp Nanocomposites on HeLa Cells

HeLa cells were used to evaluate the toxicity of lead ions. The effect of CD-HAp nanocomposites (before and after removal of lead ions from contaminated solutions) on HeLa cells (~104 cells/100 µL) viability was established by the conventional 3-(4,5-dimethylthiazolyl-2)-2,5-diphenyltetrazolium bromide (MTT) reduction assay as described in our previous reported studies [39,40]. HeLa cells were treated with CD-HAp nanocomposites before and after removal of lead ions from contaminated solutions. The effects on the cell viability were evaluated after 24 h of incubation. The HeLa cells images were taken using an Observer D1 Carl Zeiss microscope.

## 5. Conclusions

The purpose of this study was to present new and valuable information on the synthesis and physico-chemical properties, as well as ultrasonic characterization of β-Cyclodextrin/hydroxyapatite composites. Considering the concentration of β-Cyclodextrin, two different composites were obtained using an adapted co-precipitation method. Upon completion of the materials synthesis, morphological investigations were performed. The SEM study revealed that the β-Cyclodextrin/hydroxyapatite composite was made of spherical nanoparticles, which tended to agglomerate, due to their reduced size. Moreover, the particle size obtained from SEM images was in good agreement with the results acquired by DLS analysis. The EDX and elemental mapping analysis highlighted the main constituent elements (P, O and Ca) and their uniform distribution throughout the sample. In addition, Pb^2+^ was identified in the samples after lead removal from aqueous solution. Furthermore, novel ultrasonic measurements applied for the β-Cyclodextrin/hydroxyapatite composites dispersion allowed us to calculate the ultrasonic velocity and attenuation. It was observed that the ultrasonic velocity and attenuation coefficient diminished with the increase of β-Cyclodextrin concentration in the samples. Furthermore, experiments involving lead ion removal from aqueous solutions were performed. The results of our experiments showed that the initial concentration of lead from the contaminated aqueous solutions strongly influenced the adsorption ability of CD-HAp_1 and CD-HAp_2 composites. BET measurements demonstrated that the CD-HAp_1 and CD-HAp_2 nanocomposites were mesoporous. Moreover, the studies regarding the HeLa cells viability incubated with CD-HAp nanocomposites, on the one hand, highlighted the biocompatibility of nanocomposites, and, on the other hand, proved the toxicity of lead ions from contaminated solutions. Our preliminary results showed a potential application for this kind of composites for environmental purposes.

## Figures and Tables

**Table 1:** Parameters of nitrogen adsorption/desorption isotherms of CD-HAp_1 and CD-HAp_2 nanocomposites.

Sample	S_BET_ (m^2^/g)	V_p_ (nm)	D_p_ (cm^3^/g)
CD-HAp_1	102.54	0.3958	14.26
CD-HAp_2	155.71	0.4568	12.49

**Table 2:** Isotherms parameters for lead adsorption onto CD-HAp_1 and CD-HAp_2.

Pollutant	Sample	Langmuir			Freundlich			
		*q_m_*_(mg/g)_	*K_L_*_(L/mg)_	R^2^	n	1/n	*K_F_*_(mg/g (K/mg)_^1/n^_)_	R^2^
Pb^2+^	CD-HAp_1	98.232	1.378	0.99	3.088	0.324	57.971	0.981
	CD-HAp_2	105.820	2.218	0.992	2.917	0.343	75.733	0.984

**Table 3:** Parameter comparison of mean particle size resulting from DLS, SEM studies and ultrasonic measurements.

Sample	US (m^2^/g)	SEM (nm)	DLS (nm)
CD-HAp_1	23.5 ± 2	20.8 ± 1	97.7 ± 4
CD-HAp_2	16.8 ± 4	15.2 ± 2	163 ± 2
